# Recombinant CCL17-dependent CCR4 activation alleviates neuroinflammation and neuronal apoptosis through the PI3K/AKT/Foxo1 signaling pathway after ICH in mice

**DOI:** 10.1186/s12974-021-02112-3

**Published:** 2021-03-01

**Authors:** Shuixiang Deng, Peng Jin, Prativa Sherchan, Shengpeng Liu, Yuhui Cui, Lei Huang, John H. Zhang, Ye Gong, Jiping Tang

**Affiliations:** 1 Department of Critical Care Medicine, HuaShan Hospital, Fudan University, 12 middle WuLuMuQi, Shanghai, 200040 China; 2 Department of Physiology and Pharmacology, Center for Neuroscience Research, Loma Linda University School of Medicine, Risley Hall, Room 219, 11041 Campus Street, Loma Linda, CA 92350 USA; 3 Department of Pediatrics, Shenzhen People’s Hospital, The Second Clinical Medical College of Jinan University, Shenzhen, Guangdong China; 4 Department of Neurosurgery, Loma Linda University School of Medicine, Loma Linda, CA 92350 USA; 5 Department of Anesthesiology, Loma Linda University School of Medicine, Loma Linda, CA 92350 USA; 6 Department of Neurosurgery, Huashan Hospital, Fudan University, Shanghai, 200040 China

**Keywords:** C-C Chemokine Receptor 4, Recombinant CCL17, Neuroinflammation, Apoptosis, PI3K/AKT/Foxo1 signaling, Intracerebral hemorrhage

## Abstract

**Background:**

Intracerebral hemorrhage (ICH), a devastating subtype of stroke, is associated with high mortality and morbidity. Neuroinflammation is an important factor leading to ICH-induced neurological injuries. C-C Chemokine Receptor 4 (CCR4) plays an important role in enhancing hematoma clearance after ICH. However, it is unclear whether CCR4 activation can ameliorate neuroinflammation and apoptosis of neurons following ICH. The aim of the present study was to examine the effects of recombinant CCL17 (rCCL17)-dependent CCR4 activation on neuroinflammation and neuronal apoptosis in an intrastriatal autologous blood injection ICH model, and to determine whether the PI3K/AKT/Foxo1 signaling pathway was involved.

**Methods:**

Two hundred twenty-six adult (8-week-old) male CD1 mice were randomly assigned to sham and ICH surgery groups. An intrastriatal autologous blood injection ICH model was used. rCCL17, a CCR4 ligand, was delivered by intranasal administration at 1 h, 3 h, and 6 h post-ICH. CCL17 antibody was administrated by intraventricular injection at 1 h post-ICH. C021, a specific inhibitor of CCR4 and GDC0068, an AKT inhibitor were delivered intraperitoneally 1 h prior to ICH induction. Brain edema, neurobehavioral assessments, western blotting, Fluoro-Jade C staining, terminal deoxynucleotidyl transferase dUTP nick end labeling, and immunofluorescence staining were conducted.

**Results:**

Endogenous expression of CCL17 and CCR4 were increased following ICH, peaking at 5 days post-induction. CCR4 was found to co-localize with microglia, neurons, and astrocytes. rCCL17 treatment decreased brain water content, attenuated short- and long-term neurological deficits, deceased activation of microglia/macrophages and infiltration of neutrophils, and inhibited neuronal apoptosis in the perihematomal region post-ICH. Moreover, rCCL17 treatment post-ICH significantly increased the expression of CCR4, PI3K, phosphorylated AKT, and Bcl-2, while Foxo1, IL-1β, TNF-α, and Bax expression were decreased. The neuroprotective effects of rCCL17 were reversed with the administration of C021 or GDC0068.

**Conclusions:**

rCCL17-dependent CCR4 activation ameliorated neurological deficits, reduced brain edema, and ameliorated neuroinflammation and neuronal apoptosis, at least in part, through the PI3K/AKT/Foxo1 signaling pathway after ICH. Thus, activation of CCR4 may provide a promising therapeutic approach for the early management of ICH.

**Supplementary Information:**

The online version contains supplementary material available at 10.1186/s12974-021-02112-3.

## Background

With a high mortality and morbidity rate, spontaneous intracerebral hemorrhage (ICH) is considered to be catastrophic, accounting for approximately 10–20% of stroke cases [1–3]. Despite advances in the understanding of pathophysiological mechanisms of early brain injury after ICH, to date, there is no specific therapeutic approach to ICH-induced brain injury [4–6]. The mass effect of intracerebral hematoma as well as red blood cell debris and degradation products are considered to be key factors leading to early brain injury following ICH [7–9]. As a consequence, a complex inflammatory cascade leads to brain edema, blood–brain barrier (BBB) disruption, neuronal apoptosis in the perihematoma area, and other unfavorable outcomes [10, 11]. Therefore, the development of therapies that target anti-inflammatory and anti-apoptotic pathways may be crucial to improving neurological outcomes following ICH.

Chemokines and chemokine receptors play a critical role in orchestrating cell migration and homing, as well as homeostasis in the body [12]. Chemokines signal via activation of members of the seven-transmembrane G-protein-coupled receptor superfamily [12], CC chemokine ligand 17 (CCL17), a specific ligand of CC chemokine receptor 4 (CCR4), is expressed at elevated levels in many inflammatory conditions, and is induced by cytokines such as tumor necrosis factor-α (TNF-α) and interleukin-6 (IL-6) by microglia, neurons, and inflammatory myeloid cells [13, 14]. CCR4 was first discovered in a human basophilic cell line, and later shown to be abundantly expressed in natural killer cells, regulatory T cells (Tregs), monocytes, microglia, neurons, and astrocytes [15, 16]. Although CCL17 and CCR4 have been widely implicated in regulating immune and inflammatory responses [17], the expression and possible role of CCL17/CCR4 in neuroinflammation following ICH have not been examined.

The activation of many cell surface receptors, including CCR4, results in the activation of phosphoinositide 3-kinase (PI3K) pathway leading to the activation of protein kinase B/AKT and other downstream proteins associated with cell proliferation and survival [18]. The PI3K/AKT pathway is also involved in several cellular processes in pathological conditions including neurological diseases [19]. The activation of PI3K/AKT signaling pathway has been shown to alleviate brain damage and inhibit neuroinflammation and neuronal apoptosis [20]. Forkhead transcription factor 1 (Foxo1), which is necessary for pro-inflammatory cytokine production, is suppressed by phosphorylated AKT [21]. PI3K/AKT signaling is recognized as a downstream target pathway after CCR4 activation [22, 23]. However, whether PI3K/AKT/Foxo1 signaling is associated with the CCR4-mediated inflammatory reaction requires further elucidation.

In this study, we show that rCCL17-dependent CCR4 activation could ameliorate neurological deficits as well as alleviate neuroinflammation and neuronal apoptosis through the PI3K/AKT/Foxo1 signaling pathway after induction of ICH in mice.

## Materials and methods

### Animals

We housed 226 male CD1 mice (weighing 30–40 g, 8 weeks old, Charles River, Wilmington, MA) in a temperature and humidity-controlled room with a standard 12 h light/dark cycle with free access to food and water. All experimental regulations were performed according to the guidelines of National Institutes of Health for the Use and Care of Experiment Animals. In addition, the study was approved by the Institutional Animal Care and Use Committee (IACUC) at Loma Linda University. Mice were distributed to each experimental group at random via random numbers generated by Excel 10.0 software and a unique code was applied to the individual mice.

### Experimental design

All experimental animals were randomly assigned to six experimental groups and subjected to the following six experimental protocols shown in additional supplement file (Supplementary Figure S1). Surgeries, histological outcomes, and neurobehavioral testing were carried out in a blinded manner.

#### Experiment 1

To examine the dynamic changes in endogenous CCL17 and CCR4 expression over time post-ICH, 42 animals were randomly assigned to seven groups (*n* = 6/group): sham, 6 h, 12 h, 24 h, 72 h, 5 days, and 7 days post-ICH for western blot analysis. An additional four mice (*n* = 2/group) were assigned to the 72 h post-ICH and sham groups, and used to examine CCR4 localization in microglia, neurons, and astrocytes by a double immunofluorescence staining.

#### Experiment 2

To examine the role of endogenous CCL17 and CCR4 following ICH, 30 animals were randomly assigned to five groups (*n* = 6/group): sham, ICH + control mAb (150ug), ICH + anti-CCL17 mAb (150ug), ICH + C021 (1mg/kg), and ICH + DMSO. Control and anti-CCL17 mAbs were administered by intraventricular injection 1 h after ICH induction. A specific inhibitor of CCR4, C021, was delivered by intraperitoneal (i.p.) administration 1 h prior to ICH induction. Samples were collected 72 h post-ICH and changes in CCL17, CCR4, TNF-α, and interleukin 1-beta (IL-1β) protein expression levels were measured by western blot analysis.

#### Experiment 3

To examine the therapeutic window of rCCL17 following ICH, 60 animals were randomly assigned to five groups (*n* = 12/group): sham, ICH + vehicle, ICH + rCCL17 (30 μg/kg, 1 h), ICH + rCCL17 (30 μg/kg, 3 h), and ICH + rCCL17 (30 μg/kg, 6 h). Recombinant CCL17 (rCCL17) was administered intranasally (i.n.) at either 1 h, 3 h, or 6 h post-ICH. Neurological tests were conducted at 24 h and 72 h post-ICH (*n* = 6/group). Brain edema was determined at 72 h post-ICH (*n* = 6/group).

#### Experiment 4

In order to determine the effect of rCCL17 treatment on activation of microglia/macrophages, infiltration of IL-1β and neutrophils, and neuronal apoptosis at 72 h post-ICH, 30 animals were randomly subjected to three groups: sham, ICH + vehicle, and ICH + rCCL17 (30 μg/kg, 1 h). Immunofluorescence, Fluoro-Jade C (FJC) staining, and terminal deoxynucleotidyl transferase dUTP nick end labeling (TUNEL) staining were performed (*n* = 4/group). The expression of lonizing calcium-binding adaptor molecule 1 (Iba-1), myeloperoxidase (MPO), IL-1β, Bax, and Bcl-2 were determined by western blot analysis at 72 h following ICH (*n* = 6/group).

#### Experiment 5

To evaluate the long-term neurobehavioral outcome of rCCL17 treatment post-ICH, 24 animals were randomly assigned to three groups: sham, ICH + vehicle, and ICH + rCCL17 (30 μg/kg, 1 h) (*n* = 8/group). rCCL17 was administered i.n. at 1 h, 25 h, and 49 h following ICH. The Rotarod and foot fault test were conducted on days 7, 14, and 21 post-ICH. The Morris water maze test was conducted on days 22–27 following ICH.

#### Experiment 6

To examine the potential role of the CCR4/PI3K/AKT/Foxo1 signaling pathway, 36 animals were assigned to six groups: sham, ICH + vehicle (DMSO), ICH + rCCL17 (30 μg/kg, 1 h), ICH + rCCL17 (30 μg/kg, 1 h) + C021 (1mg/kg), ICH + rCCL17 (30 μg/kg, 1 h) + DMSO, and ICH + rCCL17 (30 μg/kg, 1 h) + GDC0068 (50mg/kg) (*n* = 6/group). Neurobehavioral function tests and western blot analysis were carried out at 72 h following ICH.

### ICH model

ICH surgery was performed using an intrastriatal autologous blood injection model using a stereotactic guide as previously described [24]. First, the animals were anesthetized using a mixture of ketamine (100 mg/kg) and xylazine (10 mg/kg, 2:1 vol/vol, i.p.). Then, the mice were positioned prone on the stereotactic frame (Kopf Instruments, Tujunga, CA, USA). A 1 mm hole was drilled in the skull, and a 1 mL syringe was inserted into the right basal ganglia in accordance with the stereotactic guide (0.2 mm anterior, 2.3 mm right lateral, and 3.5 mm below the dura). Following this, 30 μL autologous blood was injected using an infusion pump (Harvard Apparatus, Holliston, MA, USA). First, 5 μL of blood was administrated at a speed of 3 μL/min, and after waiting for 5 min, the remaining 25 μL blood was injected into the right striatum. To prevent leakage due to blood backflow, we waited for additional 10 min before removing the needle. Rectal temperature of the animals was monitored and maintained at 37.0 ± 0.5 °C by a surgical heating pad during surgery and recovery periods. The sham surgery was performed with needle insertion exclusively—that is, without blood injection.

### Drug administration

I.n. administration of rCCL17 was performed at 1 h post-ICH as described previously [8]. Mice were put in a supine position after deep anesthesia, then, rCCL17 diluent in DMSO or DMSO (a total volume of 20 μL) was delivered into bilateral nares with 5 μL per naris every 5 min over a period of 20 min alternating between the two nares. C021 (3581, Tocris, USA) (1 mg/kg) [25] and GDC0068 (S2808, Selleckchem, USA) (50 mg/kg) [26] were dissolved in 5% DMSO and delivered i.p. at 1 h prior to ICH. Anti-CCL17 mAb (MAB529, R&D Systems, USA) or isotype control mAb (MAB002, R&D Systems) were delivered by the intracerebroventricular (i.c.v.) route 1 h post-ICH using a microinfusion pump at a speed of 0.25 μL/min at the following position: 0.3 mm posterior, 1 mm right lateral, and 2.3 mm ventral.

### Short-term neurobehavior assessment

The corner turn test, forelimb placement test, and modified Garcia test were used to assess short-term neurological deficits at 24 h and 72 h post-ICH as previously described [8, 27]. For the corner turn test, mice were placed in a corner with a 30° angle that had access to an exit on both the right and left sides. Ten trials were recorded and a score was counted as the number of left turns/10 trials × 100%. For the forelimb placement test, the percentage of left forelimb placement was calculated from ten trials. The modified Garcia test with a 21-point score was conducted to evaluate spontaneous activity, axial sensation, symmetry of limb movement, vibrissae proprioception, forelimb walking, lateral turning and grabbing, and climbing.

### Brain water content measurement

The wet/dry method was used to measure brain water content (BWC) as previously described [28]. The animals were euthanized with isoflurane, and the brains were removed immediately and cut into five sections: ipsilateral and contralateral basal ganglia, ipsilateral and contralateral cortex, and cerebellum. Each brain section was measured on an analytical microbalance to obtain the wet weight (WW). Subsequently, each part of the brain was baked at 100 °C for 48 h to obtain the dry weight (DW). The BWC (%) was measured using the following formula: (WW−DW)/WW × 100%.

### Long-term neurobehavioral assessment

Sensorimotor coordination and balance were assessed using foot fault and Rotarod tests, which were conducted at weeks 1, 2, and 3 after ICH surgery. Memory and spatial learning abilities were tested using the Morris water maze test, which was conducted at days 22–27 post-ICH as previously described [8].

### Immunofluorescence staining

Double immunofluorescence staining was performed at 72 h post-ICH as described previously [29]. Briefly, animals were deeply anesthetized and perfused with 200 mL PBS followed by 50 mL 10% formalin transcardially. Brain samples were removed immediately and fixed in 10% formalin for 2 days. Then, 30% sucrose was used to dehydrate the brain for a further 3 days. Brain sections were prepared as 10 μm coronal slices with a cryostat (CM3050S, Leica Biosystems, USA). Samples were co-incubated with primary antibodies at 4 °C overnight: anti-Iba-1 (1:100, ab178847, Abcam, MA, USA); anti-GFAP (1:200, ab16997, Abcam); anti-NeuN (1:200, ab177487, Abcam); anti-CCR4 (1:100, Santa Cruz Biotechnology, USA); anti-myeloperoxidase (MPO) (1:200, Abcam); and anti-IL-1β (1:200, Abcam). Slides were incubated with the appropriate secondary antibodies (1:200, Jackson ImmunoResearch, USA) for 2 h at room temperature and observed using a fluorescence microscope (Leica Microsystems, USA).

### Western blotting analysis

At each post-ICH time point, the brain samples were collected and prepared for western blot analysis as previously described [8]. Briefly, the ipsilateral/right brain hemisphere sample was prepared using RIPA lysis buffer (sc-24948, Santa Cruz Biotechnology). Then, 4 μL of protein sample was loaded onto an SDS-PAGE gel and transferred to a nitrocellulose membrane. The membrane was blocked with 5% non-fat milk and incubated with the following primary antibodies: rabbit anti-CCR4 (1:1000, GTX53474, Gene Tex, USA); rabbit anti-CCL17 (1:1000, ab182793, Abcam); rabbit anti-PI3K (1:1000, Cell Signaling, Danvers, MA, USA); rabbit anti-AKT (1:1000, Cell Signaling); rabbit anti-phosphorylated AKT (p-AKT, 1:1000, Cell Signaling); rabbit anti-Foxo1 (1:1000, ab179450, Abcam); goat anti-Iba-1 (1:1000, ab5076, Abcam); mouse anti-MPO (1:1000, sc-390109, Santa Cruz Biotechnology); rabbit anti-IL-1β (1:1000, ab9722, Abcam); rabbit anti-TNF-α (1:1000, ab6671, Abcam); anti-Bcl-2 (1:2000, Abcam); anti-Bax (1:4000, Abcam); and mouse anti-β-actin (1:3000, sc-47778, Santa Cruz Biotechnology) overnight at 4 °C. Membranes were incubated with corresponding secondary antibody (1:3000, Santa Cruz; 1:5000, Abcam) for 2 h at room temperature. The relative density of the protein bands was quantified by the ImageJ software (ImageJ 1.4, NIH, Bethesda, MD, USA).

### TUNEL staining

According to the manufacturer’s instructions, double staining of neuron marker NeuN (green) and TUNEL (red) was applied to quantify neuronal apoptosis with in situ Apoptosis Detection Kit (Roche, Indianapolis, USA) at 72 h post-ICH. The number of TUNEL-positive neurons was analyzed in the perihematomal region. Four random visual fields per slide were averaged by a blinded observer using a microscope at ×400 magnification. Data were measured by the ratio of TUNEL-positive neurons (%).

### FJC Staining

The number of degenerating neurons was assessed by FJC staining using a modified FJC Ready-to-Dilute Staining Kit (Millipore, Billerica, MA, USA) at 72 h post-ICH as previously described [30]. In accordance with the manufacturer’s instructions, slides were washed with PBS incubated with the FJC working solution for 20 min, and then visualized using a fluorescence microscope (Leica Microsystems). The FJC-positive neurons in four parts of the perihematomal area of each brain were manually counted using a microscope at ×400 magnification and the ImageJ software (ImageJ 1.5, NIH, USA). Data were calculated and presented as the number of positive cells per mm^2^.

### Statistical analysis

All data are presented as the mean ± standard deviation (mean ± SD). Western blot analysis used one-way ANOVA followed by Tukey’s post hoc test. Neurological functions were analyzed by two-way ANOVA and Tukey’s post hoc test, and *p* < 0.05 was considered to be statistically significant. All data were analyzed by GraphPad Prism 7 (San Diego, CA, USA).

## Results

### Animal use

A total of 226 mice were used in this study with 176 animals assigned to ICH surgery. No mice died during the sham-operated procedure. In the present study, a total of 5 mice died during surgery. Two mice died before ICH induction due to anesthesia, one mouse died during ICH model surgery, and two mice died during intraventricular injection administration. The total mortality rate in the study was 2.84% (5/176). There were no significant differences in the mortality rate among all ICH experimental groups. No mice were excluded from the study.

### The expression of CCL17 and CCR4 were enhanced at the acute-term after ICH

**Fig. 1:**
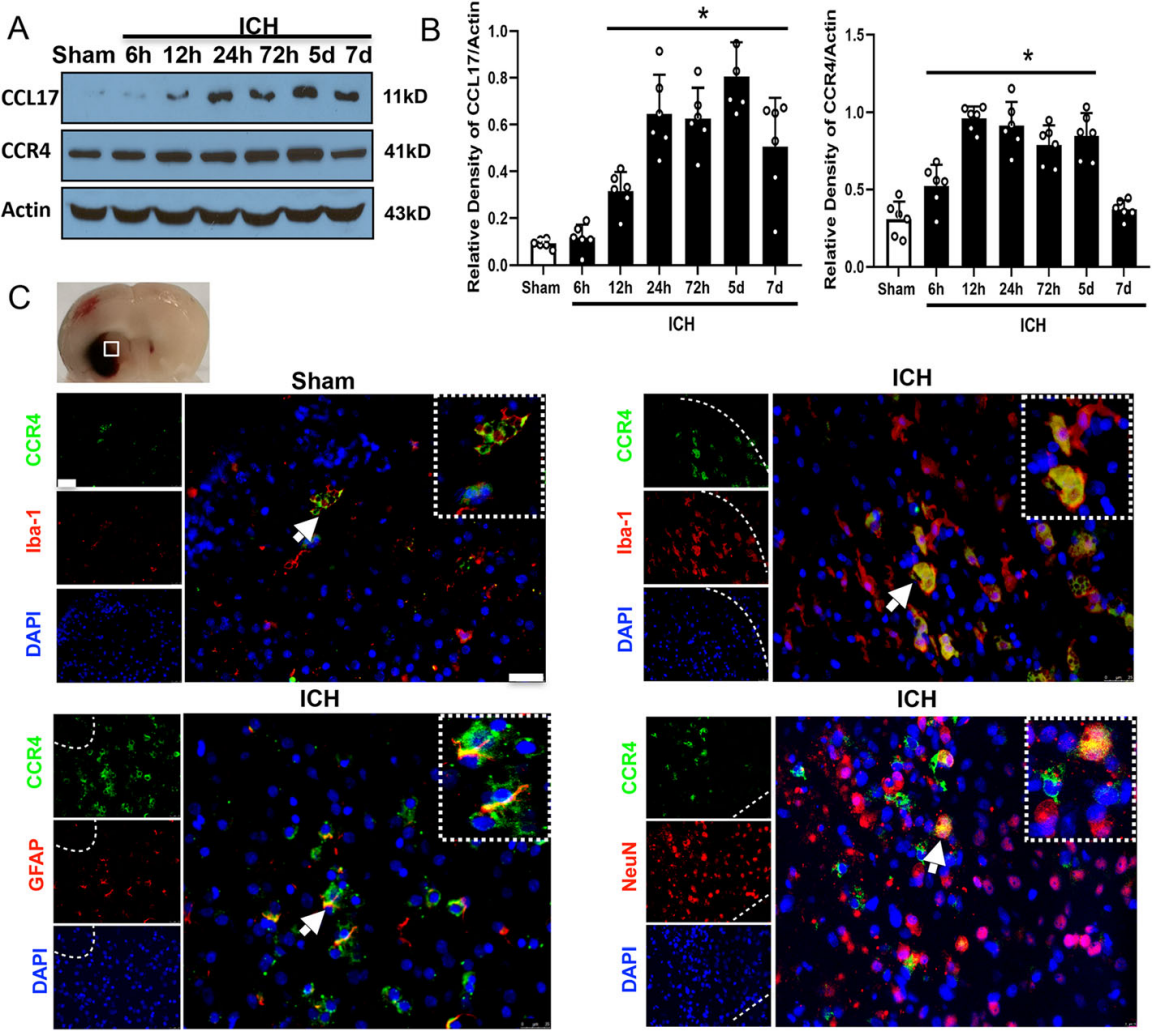
Expression profile of CCL17 and CCR4 after ICH. **a** Representative western blot bands of time course and (**b**) quantitative analysis of CCL17 and CCR4 expressions, respectively after ICH; *p<0.05 vs sham, mean ± SD, one-way ANOVA, Tukey test, *n*=6/group. **c** Brain sample with schematic illustration showing the area in perihematomal region (indicated by white box) from where the images were taken for immunofluorescence staining. Representative images of co-localization of CCR4 (green) with microglia/macrophage (Iba-1, red), astrocytes (GFAP, red), and neurons (NeuN, red) in the perihematomal area at 72 h after ICH (the dotted white line shows the hematoma area). Nuclei were stained with DAPI (blue), scale bar=50 μm, *n*=2/group, DAPI, 4′,6-diamidino-2-phenylindole

Dynamic changes in CCL17 and CCR4 expression levels were evaluated by western blot analysis at 0 (sham), 6 h, 12 h, 24 h, 72 h, 5 days, and 7 days post-ICH. CCL17 protein expression was markedly increased at 24 h, peaked at day 5, then decreased at day 7 post-ICH compared with the sham group (*p* < 0.05, Fig. 1a, b). A significant increase in CCR4 expression was observed between 12 h to 5 days post-ICH in the ICH group compared to the sham group (*p* < 0.05, Fig. 1a, b). Immunohistochemical staining of CCR4 revealed that CCR4 was expressed on microglia, neurons, and astrocytes in the perihematomal area at 72 h post-ICH (Fig. 1c).

### Endogenous CCL17 and CCR4 attenuated neuroinflammation post-ICH

**Fig. 2:**
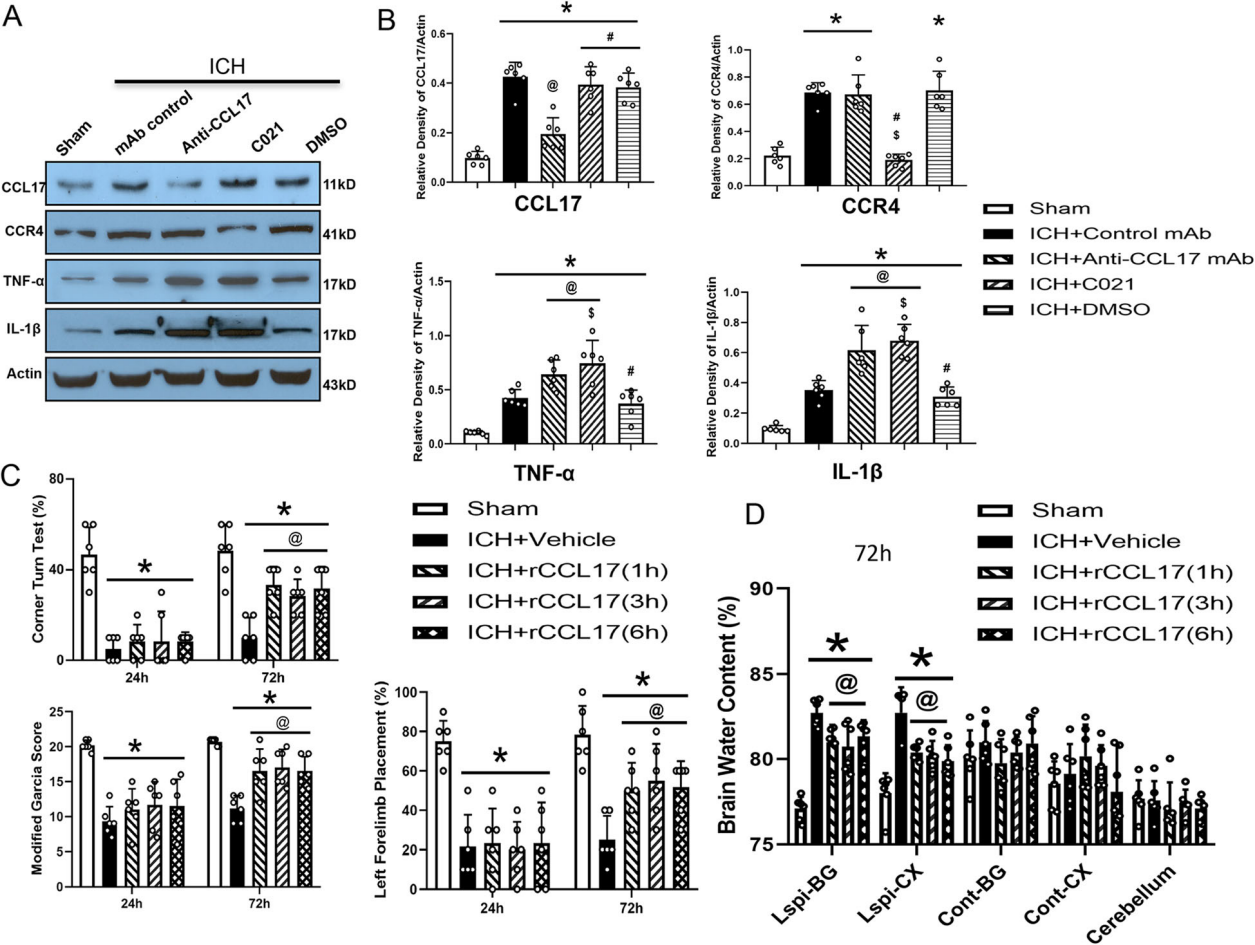
To evaluate the role of endogenous CCL17 and CCR4 after ICH. Endogenous CCL17 and CCR4 increased at 72 h after ICH. Anti-CCL17 mAb and C021 abolished the effect of endogenous CCL17 and CCR4 and increased inflammatory markers at 72 h after ICH (**a**, **b**). Intranasal administration of rCCL17 (1 h, 3 h, and 6 h) improved neurological function. *p<0.05 vs sham, @*p*<0.05 vs ICH + control mAb, #*p*<0.05 vs ICH + Anti-CCL17 mAb, $*p*<0.05 vs ICH +DMSO, One-way ANOVA, Tukey’s test, *n*=6/group. **c** Behavior tests. Left forelimb placement test (**c**), Corner turn test(**c**) and Modified Garcia test (**c**). **d** Brain water content. *p<0.05 vs sham, @*p*<0.05 vs vehicle, one-way ANOVA, Tukey’s test, *n*=6/group.

Endogenous CCL17 and CCR4 protein levels were significantly higher in the ICH group compared to the sham group 72 h post-ICH (*p* < 0.05, Fig. 2a, b). Treatment with anti-CCL17 mAb or the CCR4 inhibitor, C021, led to increased expression of the proinflammatory cytokines, TNF-α, and IL-1β, in both the ICH + anti-CCL17 mAb and ICH + C021 groups compared to the ICH + control mAb and ICH + DMSO groups (*p* < 0.05, Fig. 2a, b).

### rCCL17 treatment at different time points (1 h, 3 h, and 6 h) improved short-term neurological function and alleviated BWC at 72 h post-ICH

In order to evaluate the therapeutic window of rCCL17 after ICH induction, rCCL17 was administered at either 1 h, 3 h, or 6 h after ICH. The acute neurological function score was markedly decreased while the BWC in ipsilateral basal ganglia and cortex was markedly augmented in all ICH groups compared to the sham group at 24 h and 72 h post-ICH (*p* < 0.05, Fig. 2c, d). Administration of rCCL17 at 1 h, 3 h, and 6 h significantly attenuated neurobehavioral deficits and BWC at 72 h post-ICH compared to the ICH + vehicle group (*p* < 0.05, Fig. 2c, d). Based on these results, rCCL17 administration at 1 h post-ICH was selected for further studies.

### rCCL17 treatment attenuated neuroinflammation at 72 h post-ICH

**Fig. 3:**
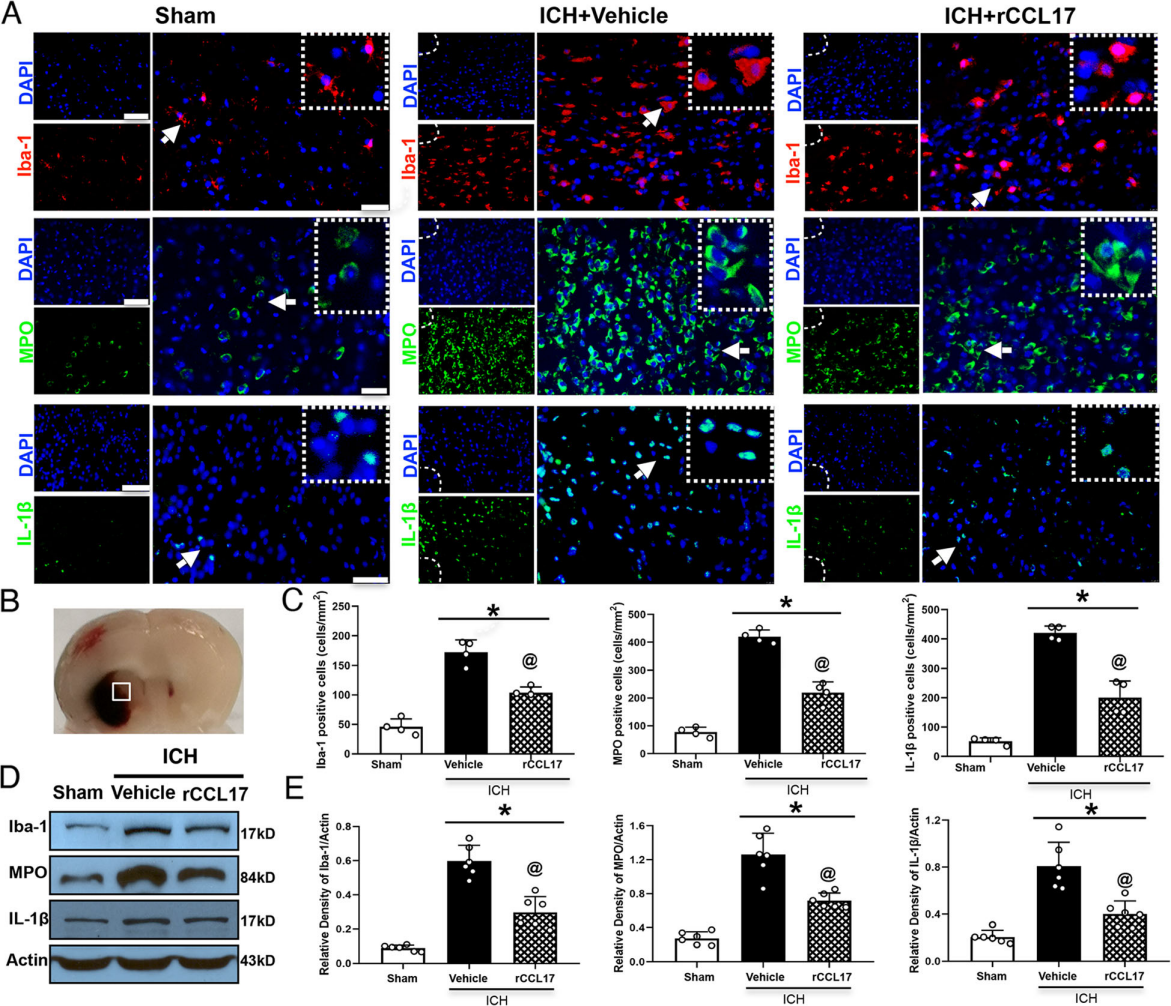
The effects of rCCL17 treatment on microglia/macrophage activation and neutrophil infiltration after ICH. **a** Representative images of immunofluorescence staining of Iba-1 (red), MPO (green), and IL-1β (green) in the perihematomal area at 72 h after ICH. **b** Brain sample with schematic illustration showing the area in the perihematomal region (indicated by white box) from where the images were taken for immunofluorescence staining. **c** Quantitative analyses of Iba-1, MPO and IL-1β positive cells in the perihematomal area at 72 h after ICH (*n*=4/group). **d**, **e** Representative western blot bands and quantitative analyses of Iba-1, MPO, and IL-1β protein levels at 72 h after ICH. *p<0.05 vs sham, @*p*<0.05 vs ICH + vehicle, mean ± SD, one-way ANOVA, Tukey test, *n*=6/group, scale bar=50 μm

Immunofluorescence staining and western blot analysis were conducted to examine the expression of Iba-1, IL-1β, and MPO at 72 h post-ICH. Immunofluorescence staining results showed that microglia/macrophage activation and neutrophil infiltration were markedly increased in the ICH + vehicle group. However, compared to sham-treated mice, rCCL17 administration significantly reduced the number of Iba-1-, MPO-, and IL-1β-positive cells in the ICH + vehicle group in the perihematomal region at 72 h post-ICH (*p* < 0.05, Fig. 3a-c). Similarly, western blot analysis revealed that Iba-1, MPO, and IL-1β protein expression levels were significantly decreased in the ipsilateral hemisphere of the rCCL17-treatment group compared to the ICH + vehicle group (*p* < 0.05, Fig. 3d, e).

### rCCL17 administration alleviated neuronal apoptosis at 72 h post-ICH

**Fig. 4:**
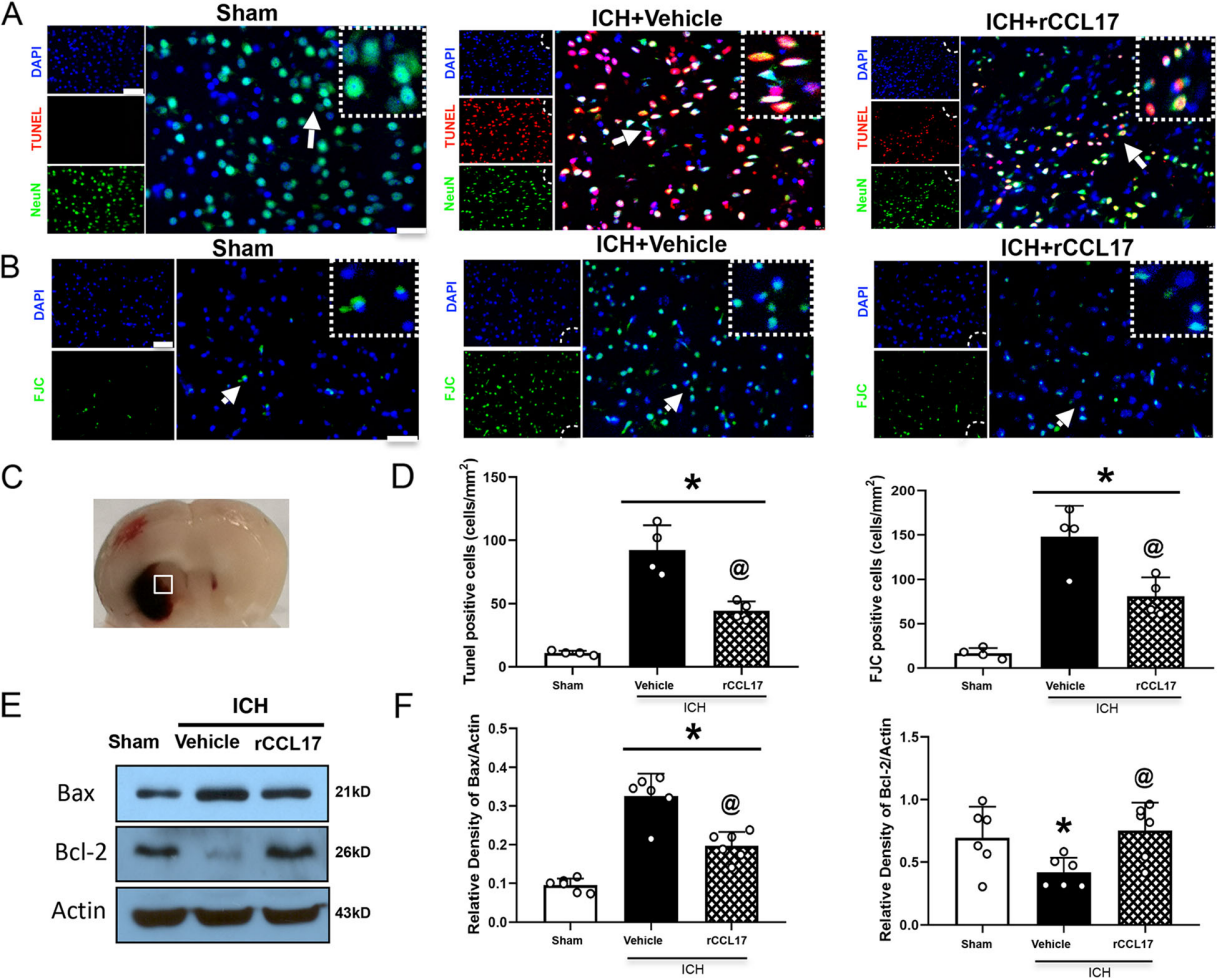
The effects of rCCL17 treatment on neuronal apoptosis and neuronal apoptotic molecular markers after ICH. **a** Representative images of the co-localization of TUNEL (red) with neurons (NeuN, green) and (**b**) FJC (green) staining in the perihematomal area at 72 h after ICH. **c** Brain sample with schematic illustration showing the area (indicated by white square) used for FJC and TUNEL positive cell counting in the perihematomal region. **d** Quantitative analyses of TUNEL and FJC-positive cells in the perihematomal area at 72 h after ICH (*n*=4/group). **e**, **f** Representative western blot bands and quantitative analyses of Bax and Bcl-2 protein levels at 72 h after ICH. *p<0.05 vs sham, @*p*<0.05 vs ICH + vehicle, mean ± SD, one-way ANOVA, Tukey test, *n*=6/group, scale bar=50 μm

FJC and TUNEL staining, and western blot analysis were chosen to evaluate neuronal degeneration and apoptosis in the perihematomal region at 72 h post-ICH. TUNEL-positive and FJC-positive neurons were significantly increased in the perihematomal region of the vehicle-treated ICH group compared to the sham group at 72 h post-ICH. However, rCCL17 administration decreased the number of TUNEL-positive and FJC-positive neurons (*p* < 0.05, Fig. 4a-d). Consistent with these findings, western blot analysis revealed that rCCL17 treatment in the ipsilateral hemisphere led to a significant increase in Bcl-2 (pro-survival marker) expression, while Bax (pro-apoptotic marker) levels were markedly reduced compared to the vehicle-treated ICH group at 72 h post-ICH (*p* < 0.05, Fig. 4e, f).

### rCCL17 treatment improved long-term neurobehavioral function post-ICH

**Fig. 5:**
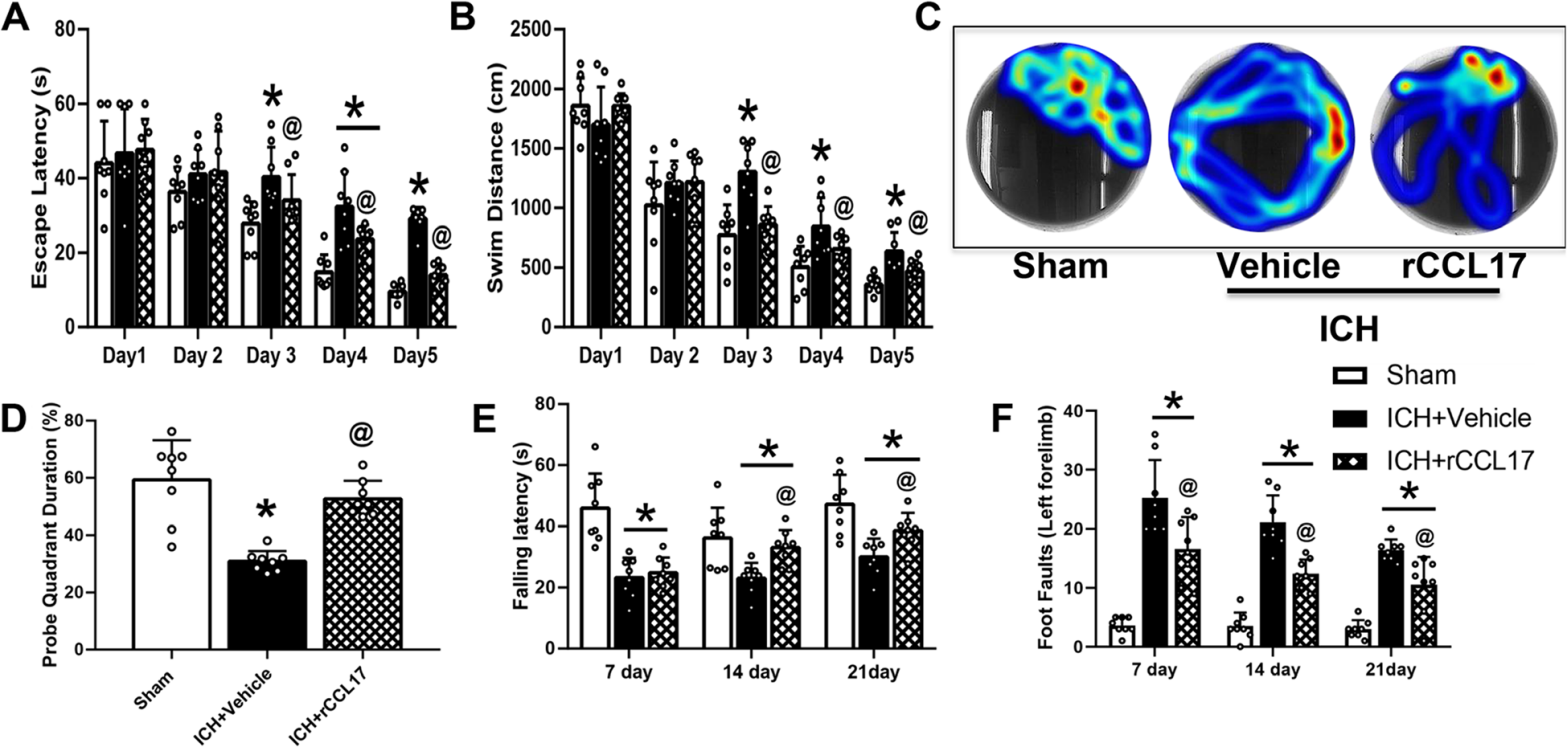
rCCL17 treatment improved long-term neurological function after ICH. **a** Escape latency and (**b**) swim distance in Morris water maze testing on days 21 to 25 after ICH. **c** Typical traces of Morris water maze test on day 27 after ICH. **d** Probe quadrant duration in Morris water maze test on day 27 after ICH. **e** Foot fault test and (**f**) Rotarod test performed at days 7, 14, and 21 after ICH. *p<0.05 vs sham, @*p*<0.05 vs ICH + vehicle, mean ± SD, one-way ANOVA, Tukey test, *n*=8/group

The Morris water maze test revealed that the escape latency and swim distance in ICH-induced mice were significantly increased compared to the sham group (*p* < 0.05, Fig. 5a, b, c). rCCL17 treatment markedly reduced the escape latency and swim distance on testing days 3 to 5 when compared with vehicle-treated ICH group (*p* < 0.05, Fig. 5a, b, c). The probe trial revealed that mice in the ICH + vehicle group spent markedly less time in the target quadrant, while rCCL17-treated mice spent significantly longer time in the probe quadrant compared with the ICH + vehicle group (Fig. 5d). However, in the Rotarod and foot fault tests, vehicle-treated mice had a markedly shorter falling latency and significantly increased left forelimb foot-faults compared to the sham group at days 7, 14, and 21 post-ICH (*p* < 0.05; Fig. 5e, f). rCCL17 administration ameliorated these neurological deficits in both tests when compared with the ICH + vehicle group (*p* < 0.05; Fig. 5e, f).

### rCCL17 treatment alleviated neuroinflammation and neuronal apoptosis via the CCR4/PI3K/AKT/Foxo1 signaling pathway at 72 h post-ICH

**Fig. 6:**
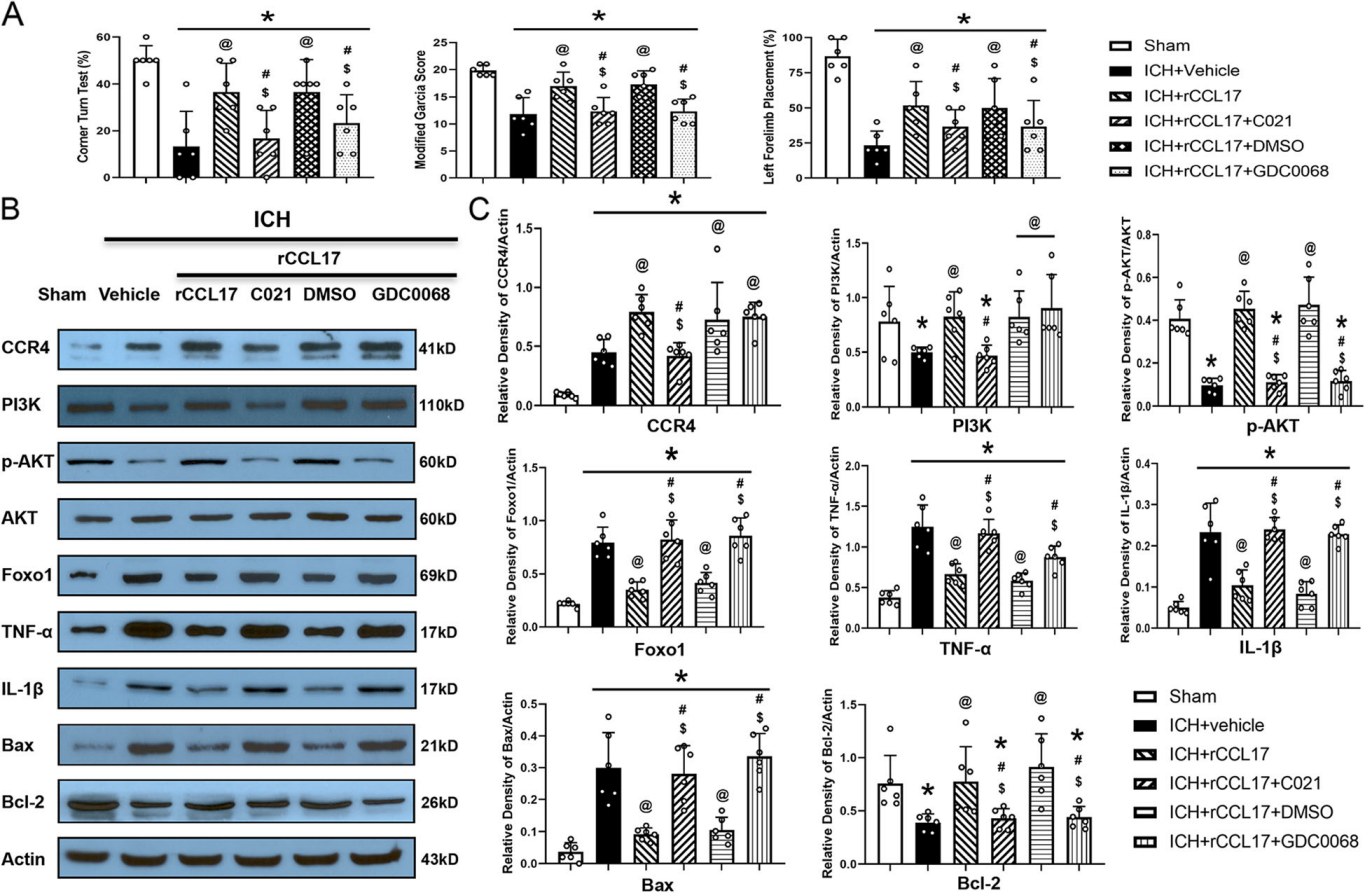
Inhibition of CCR4 and AKT reversed the effects of rCCL17 on neurological functions and inflammatory/apoptotic proteins expression. **a** Modified Garcia test, corner turn, test and forelimb placement test of each group at 72 h after ICH. **b**, **c** Representative western blot bands and quantitative analyses of CCR4, PI3K, phosphorylated AKT (p-AKT), AKT, Foxo1, TNF-α, IL-1β, Bax, and Bcl-2 expression in the ipsilateral hemisphere at 72 h after ICH. *p<0.05 vs sham, @*p*<0.05 vs ICH + vehicle, #*p*<0.05 vs ICH + rCCL17, $*p*<0.05 vs ICH + rCCL17+ DMSO, mean ± SD, one-way ANOVA, Tukey’s test, *n*=6/group

Pretreatment with the CCR4 inhibitor, C021, or the pan AKT inhibitor, GDC0068, significantly abolished the neurobehavioral benefits of rCCL17 in the corner turn, modified Garcia, and forelimb placement tests at 72 h post-ICH (*p* < 0.05, Fig. 6a). Western blot analysis revealed that after rCCL17 treatment, CCR4 expression was markedly upregulated compared to both the ICH + vehicle and sham groups (*p* < 0.05, Fig. 6b, c). However, CCR4 expression was significantly downregulated after C021 treatment in the ICH + rCCL17 + C021 group compared with the ICH + rCCL17 group (*p* < 0.05; Fig. 6b, c). GDC0068 treatment did not affect CCR4 expression after ICH (Fig. 6b, c).

In addition, PI3K, p-Akt, and Bcl-2 protein levels were markedly reduced, while Foxo1, TNF-α, IL-1β, and Bax protein levels were significantly upregulated in the ICH + vehicle group compared to the sham group at 72 h post-ICH (*p* < 0.05, Fig. 6b, c). rCCL17 treatment significantly increased PI3K, p-Akt, and Bcl-2 protein levels and markedly reduced Foxo1, TNF-α, IL-1β, and Bax protein levels in the ICH + rCCL17 group compared to the ICH + vehicle group at 72 h post-ICH (*p* < 0.05, Fig. 6b, c). Furthermore, administration of C021 or GDC0068 abolished the effect of rCCL17 on CCR4/PI3K/AKT/Foxo1-mediated neuroinflammation and neuronal apoptosis. These effects were associated with increased Foxo1, TNF-α, IL-1β, and Bax protein levels and reduced PI3K, p-AKT, and Bcl-2 protein levels compared with the ICH + rCCL17 and ICH + rCCL17 + DMSO groups (*p* < 0.05, Fig. 6b, c).

## Discussion

In the present study, we examined the neuroprotective effect of rCCL17-dependent CCR4 activation using an intrastriatal autologous blood injection ICH model. We found that the activation of CCR4 by rCCL17 alleviated short- and long-term neurobehavioral deficits, attenuated brain edema, deceased activation of microglia/macrophages and infiltration of neutrophils, and inhibited neuronal apoptosis in the perihematomal region following ICH. Moreover, rCCL17 treatment was associated with an increase in the expression of CCR4, PI3K, phosphorylated AKT and Bcl-2, and a decrease in the expression of Foxo1, TNF-α, IL-1β, and Bax following ICH. Furthermore, inhibition of CCR4 and AKT abolished the neuroprotective effects of rCCL17 on neurological functions and expression of pro-inflammatory mediators and apoptotic markers. Therefore, our findings suggest that rCCL17 administration may partially attenuate neuroinflammation and neuronal apoptosis associated with activation of the CCR4/PI3K/AKT/Foxo1 signaling pathway after ICH.

Chemokines, small molecular weight proteins involved in cell migration and activation, have been shown to be synthesized and secreted by different cells in the central nervous system (CNS) [31, 32]. Chemokine receptor activation of downstream signaling pathways has a vital role in immunobiology and neurobiology, and has been associated with multiple clinical diseases such as CNS autoimmunity, neurodegenerative diseases, and ischemic injuries [33–35]. CCL17 acts via the chemokine receptor CCR4. CCL17 serves as a chemoattractant, primarily involved in the recruitment of Th2 cells and CD4+ Tregs [36, 37]. CCL17 and its receptor CCR4 play a central role in the pathogenesis of endotoxin shock and inflammatory diseases such as atopic dermatitis and asthma [38, 39]. However, their effects on microglia activation and neuroinflammation after ICH remain unknown.

In the current study, we administered rCCL17 by intranasal route. It is well known that the BBB is an important factor limiting the application of drug from entering the central nervous system. Intranasal method is an easy and non-invasive method that permits the delivery of drugs to the central nervous system bypassing BBB. There is increasing evidence that support neuroprotective agents were successfully delivered to the brain via the nasal route [40]. We chose to deliver the small molecule protein rCCL17 i.n. for the sake of targeting it efficiently to the brain across the BBB.

In the present study, we found that endogenous CCL17 and CCR4 were upregulated at 24 h post-ICH, with levels peaking at day 5 post-ICH. Furthermore, we found that expression of the proinflammatory cytokines, TNF-α, and IL-1β was increased post-ICH. These findings were consistent with a previous study that found lipopolysaccharide (LPS)-mediated induction of CCL17 in the hippocampus was dependent on TNF signaling and affected the morphology and function of microglia [14]. CCR4 expression also increased in patients with traumatic brain injuries, and it has been associated with increased immunosuppressive cytokine expression via activated T-lymphocytes [41]. Interestingly, our immunofluorescence staining data confirmed that CCR4 co-localized not only with microglia but also with neurons and astrocytes after ICH. Our data are consistent with those of Cadosch et al., who demonstrated that upregulation of CCR4 after ICH led to decreased pro-inflammatory cytokine expression that afforded neuroprotection [41].

CCR4 receptor is a high-affinity receptor for its ligand CCL17. An increase in CCL17 expression may require the upregulation of CCR4 after ICH injury to carry out its function. This may be a reasonable explanation for increase in the expression of CCR4 post-ICH. In the present study, our western blot results demonstrated that rCCL17 administration increased the expression of CCR4 but pretreatment with C021 decreased the expression of CCR4. Previous study showed intraperitoneal administration of C021 diminished the levels of CCL17 [42]. In the present study, we observed that C021 reduced the expression of CCR4. We speculate that C021 decreased the production of CCL17 after ICH, and this decrease of the CCR4 ligand CCL17 led to reduced stimulation of CCR4, thus downregulating the amount and expression of CCR4 to adapt to the change. This is our speculation and needs to be confirmed by further studies.

We next sought to determine the mechanisms that regulate the upregulation of CCL17 and CCR4 after ICH. We speculated that the acute stress and secondary inflammatory cascade following ICH injury may stimulate immune cell infiltration into the brain leading to expression of CCR4 and secretion of CCL17 after ICH. In our ICH model, we found that the activation of microglia is associated with CCL17 production, leading to upregulated expression of its receptor CCR4. In addition, we found that nasal administration of rCCL17 increased CCR4 expression in the brain and improved neurobehavioral deficits and reduced brain edema following ICH. Thus, upregulation of endogenous CCL17 post-ICH may not be sufficient to effectively alleviate the neuroinflammatory response following ICH, and exogenous administration of rCCL17 may provide further neuroprotective effects against neuroinflammation following ICH.

In the current study, nasal administration of rCCL17 was used, as this is an easy and non-invasive way to bypass the BBB and deliver drugs to the brain [43]. Intranasal administration of rCCL17 can be a method to deliver a higher concentration reaching the brain due to its small molecular weight. Factors, such as these, that can impact clinical translation should be considered when developing strategies to target ICH in experimental animal models. Thus, using i.n. administration as the method of drug delivery, we evaluated the different therapeutic windows of rCCL17, and we found that rCCL17 treatment at different time points (1 h, 3 h, and 6 h) after ICH improved short-term neurological function and alleviated brain edema at 72 h post-ICH. Taken together, these findings provide sufficient theoretical basis for the potential application of this drug in clinical ICH patients.

Multiple studies have suggested that the inflammatory cascade following ICH plays a vital role in exacerbating the mass effect of hematoma, accelerating perihematomal edema, promoting neuronal apoptosis, and worsening the neurological outcome after ICH [44]. Additionally, activation of microglia/macrophages and infiltration of neutrophils post-ICH injury may further facilitate the production of proinflammatory cytokines and neuronal apoptotic pathways, which in turn lead to neuroinflammation and neuronal apoptosis [45]. Consistent with previous studies [8], our study found that rCCL17 therapy attenuated neurological deficits and decreased BWC at 72 h post-ICH. rCCL17 treatment also attenuated activation of microglia/macrophages, infiltration of neutrophils, and neuronal apoptosis, as well as downregulated of TNF-α, IL-1β, Bcl-2 and Bax expression in the perihematomal region following ICH. More importantly, rCCL17 administration ameliorated long-term spatial learning, memory, and the movement and balance ability.

In our previous study, we mainly focused on the exploration of therapeutic dose-response of CCL17 and its effect on hematoma clearance after ICH [8]. In the present study, we focused on the effect of CCR4 on neuroinflammation and apoptosis. We further explored the neuroprotective role of CCL17/CCR4 axis by exploring the therapeutic window of rCCL17 and the effects of inhibiting CCL17 and CCR4, respectively. The neuroprotective effects of rCCL17 may be due to its ability to promote hematoma resolution, which in turn leads to decreased neuronal necrosis, increased BBB integrity, and alleviated brain edema post-ICH [8]. rCCL17 treatment may also reduce the toxicity of cleavage products such as hemoglobin, thereby alleviating local inflammation and oxidative stress [8]. Finally, the anti-inflammatory and anti-apoptotic roles of rCCL17 may involve CCL17 facilitating chemotaxis by Treg cells to the brain where they can suppress the inflammatory response.

Our findings show that CCL17 and CCR4 signaling has a critical role in neuroinflammation and neuronal apoptosis. However, the potential molecular mechanisms downstream of CCR4 in ICH require further elucidation. PI3K regulates several key events in the inflammatory response to external insults [46, 47]. AKT, a downstream protein of PI3K activation, negatively regulates multiple downstream proteins such as forkhead transcription factors (FOXOs), Bcl-2, and nuclear factor-kappa B (NF-kB). These target proteins play important roles in cellular functions, including protein synthesis, survival, apoptosis, and neuroinflammatory disorders [48–50]. Previous studies have reported that PI3K/AKT-dependent suppression of Foxo1 attenuated brain injury and enhanced anti-oxidative responses that further suppressed inflammation and apoptosis [49, 51, 52].

Our data show that PI3K and p-AKT expression increased after rCCL17 treatment and CCR4 inhibition by C021. Suppression of AKT phosphorylation using the inhibitor, GDC0068, markedly abolished the anti-inflammatory and anti-apoptotic effects of rCCL17 treatment, suggesting that administration of rCCL17 orchestrates a multifaceted response to inhibit neuroinflammation and neuronal apoptosis. This response after ICH may involve, at least in part, activation of the PI3K/AKT/Foxo1 signaling pathway through CCR4. Our findings are consistent with those of previous studies [45, 47].

The present study has several limitations. First, CCR4 has a vital role in regulating innate and adaptive immunity via migration and recruitment of Tregs. In the current study, we confirmed that the anti-inflammatory and anti-apoptotic effects were due to CCR4. However, since we cannot exclude the possibility that Treg cells participated in the anti-inflammatory effect of CCR4, further studies are required. Second, our double immunofluorescence staining demonstrated that CCR4 colocalized not only with microglia but also with neurons and astrocytes following ICH. Since it is well established that astrocytes are involved in protecting the BBB from disruption and have an anti-inflammatory effect, it is possible that CCR4 expressed on astrocytes and neurons may contribute to the rCCL17-dependent effects post-ICH. Further studies are therefore required to better understand the role of CCR4 on other types of CNS cells. Finally, our data provide an associative link between CCR4 and the PI3K/AKT/Foxo1 signaling pathway in the regulation of inflammatory and apoptosis. Since the pathophysiology of neuroinflammation following ICH is a relatively complex network, future studies should consider elucidating other potential mechanisms underlying CCR4 activation-mediated neuroprotective effects against secondary brain injury after ICH. Lastly, emerging evidence indicated that sex and age are two major determining risk factors and prognosis after ICH [53, 54]. Preclinical studies showed that ICH-induced more severe brain damage in male and aged animals compared with young females [55]. In the present study, we only used 8-week-old male adult CD1 mice to determine the effects of rCCL17. We did not evaluate the effects of rCCL17 in different age groups and female animals, which is a limitation of this study. Therefore, varying age groups and females should be included in future studies to evaluate the neuroprotective effects of CCR4 activation in ICH.

## Conclusions

Our study indicated that rCCL17-dependent CCR4 activation improved neurological deficits, reduced brain edema, and ameliorated neuroinflammation and neuronal apoptosis after ICH. These responses were mediated, at least in part, through the PI3K/AKT/Foxo1 signaling pathway. These findings offer unique insights into the key molecules involved in C-C chemokine receptor signaling, and they offer a unique approach to the development of strategies targeting immune responses that regulate neuroinflammation in the early management of ICH.

## Supplementary Information

**Additional file 1: Supplement Figure 1.** Experimental design and animal groups. ICH, intracerebral hemorrhage; rCCL17, recombination C-C chemokine ligand17; WB, western blot; IF, immunofluorescence; BWC, brain water content; i.n., intranasally; i.c.v, intracerebroventricularly; i.p: intraperitoneal; DMSO: dimethylsulfoxide; FJC: Fluoro-Jade C; TUNEL: Terminal deoxynucleotidyl transferase dUTP nick end labeling.

## Data Availability

The datasets used and/or analyzed during the current study are available from the corresponding author on reasonable request.

## Abbreviations

ICH: Intracerebral hemorrhage
CCL17/TARC: Chemokine (C-C motif) ligand 17/thymus and activation-regulated
rCCL17: Recombination C-C chemokine ligand 17
CCR4: C-C Chemokine Receptor 4
PI3K: Phosphatidylinositol 3-kinase
Akt: Protein kinase B
Foxo1: Fork head transcription factors 1
MPO: Myeloperoxidase
IL-1β: Interleukin-1β
TNF-α: Tumor necrosis factor α
i.n.: Intranasal
i.p.: Intraperitoneal
DMSO: Dimethylsulfoxide
PBS: Phosphate-buffered saline
i.c.v.: Intracerebroventricular
BWC: Brain water content
WW: Wet weight
DW: Dry weight
FJC: Fluoro-Jade C
TUNEL: Terminal deoxynucleotidyl transferase dUTP nick end labeling
CNS: Central nervous system
BBB: Blood-brain barrier
NF-kB: Nuclear factor-kappa B
