# Supplementary figures and images for: Recombinant CCL17-dependent CCR4 activation alleviates neuroinflammation and neuronal apoptosis through the PI3K/AKT/Foxo1 signaling pathway after ICH in mice
Source: J Neuroinflammation. 2021 Mar 1;18:62. doi: 10.1186/s12974-021-02112-3 (PMC7923481; doi:10.1186/s12974-021-02112-3)

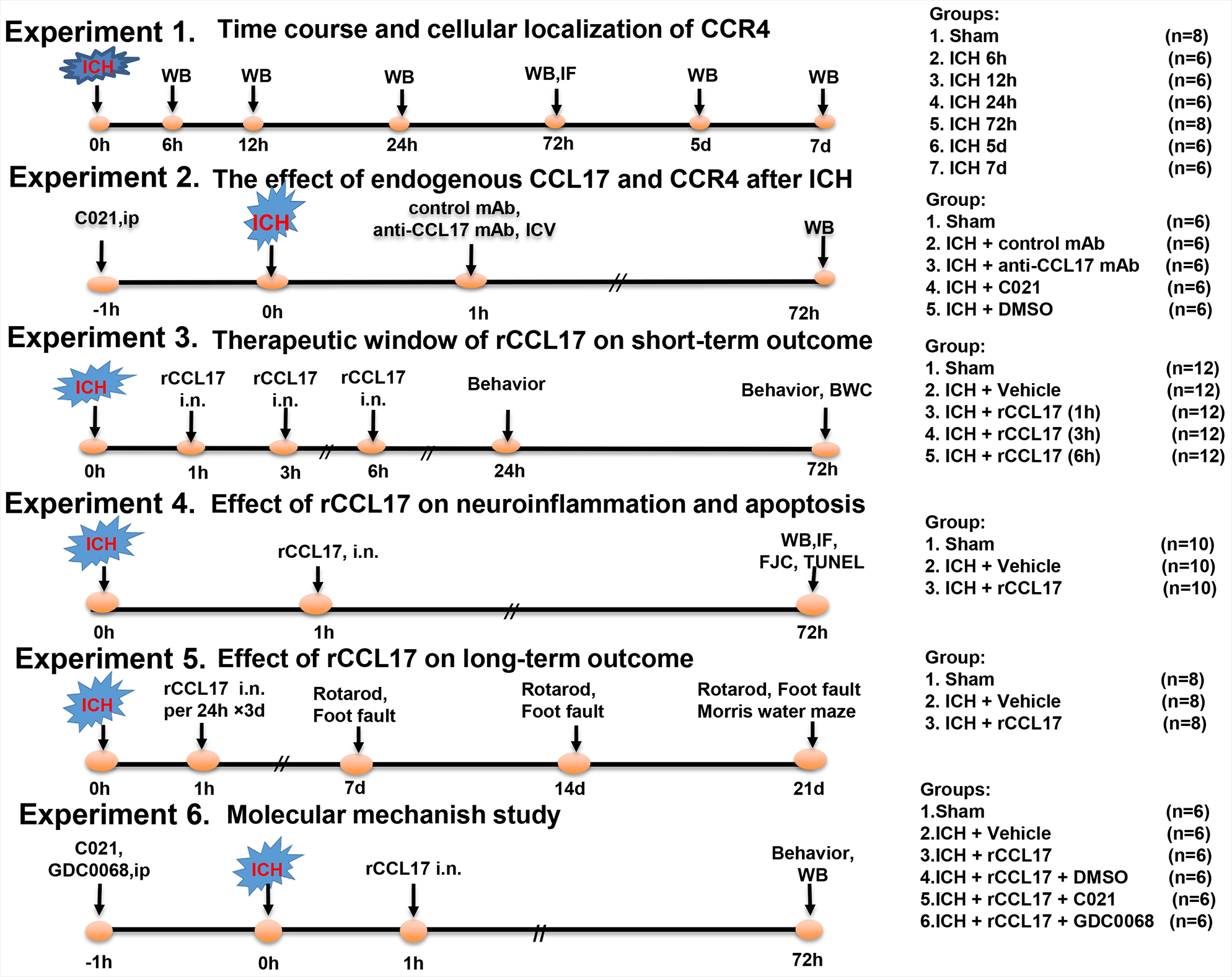

Supplement: Supplementary file 1 — Additional file 1: Supplement Figure 1. Experimental design and animal groups. ICH, intracerebral hemorrhage; rCCL17, recombination C-C chemokine ligand17; WB, western blot; IF, immunofluorescence; BWC, brain water content; i.n., intranasally; i.c.v, intracerebroventricularly; i.p: intraperitoneal; DMSO: dimethylsulfoxide; FJC: Fluoro-Jade C; TUNEL: Terminal deoxynucleotidyl transferase dUTP nick end labeling. [file 12974_2021_2112_MOESM1_ESM.tif]
